# Alcohol Enhances *Acinetobacter baumannii*-Associated Pneumonia and Systemic Dissemination by Impairing Neutrophil Antimicrobial Activity in a Murine Model of Infection

**DOI:** 10.1371/journal.pone.0095707

**Published:** 2014-04-21

**Authors:** Jay A. Gandhi, Vaibhav V. Ekhar, Melissa B. Asplund, Asan F. Abdulkareem, Mohammed Ahmadi, Carolina Coelho, Luis R. Martinez

**Affiliations:** 1 Department of Biomedical Sciences, Long Island University-Post, Brookville, New York, United States of America; 2 Department of Biology, Adelphi University, Garden City, New York, United States of America; 3 Department of Microbiology and Immunology, Albert Einstein College of Medicine, Bronx, New York, United States of America; 4 Division of Infectious Diseases, Department of Medicine, Albert Einstein College of Medicine, Bronx, New York, United States of America; 5 PhD Program in Experimental Biology and Biomedicine, Centre for Neuroscience and Cell Biology of Coimbra and Institute of Microbiology, Faculty of Medicine, University of Coimbra, Coimbra, Portugal; Albany Medical College, United States of America

## Abstract

*Acinetobacter baumannii* (*Ab*) is a common cause of community-acquired pneumonia (CAP) in chronic alcoholics in tropical and sub-tropical climates and associated with a >50% mortality rate. Using a murine model of alcohol (EtOH) administration, we demonstrated that EtOH enhances *Ab*-mediated pneumonia leading to systemic infection. Although EtOH did not affect neutrophil recruitment to the lungs of treated mice, it decreased phagocytosis and killing of bacteria by these leukocytes leading to increased microbial burden and severity of disease. Moreover, we determined that mice that received EtOH prior to *Ab* infection were immunologically impaired, which was reflected in increased pulmonary inflammation, sequential dissemination to the liver and kidneys, and decreased survival. Furthermore, immunosuppression by EtOH was associated with deregulation of cytokine production in the organs of infected mice. This study establishes that EtOH impairs immunity *in vivo* exacerbating *Ab* infection and disease progression. The ability of *Ab* to cause disease in alcoholics warrants the study of its virulence mechanisms and host interactions.

## Introduction

*Acinetobacter baumannii* (*Ab*) is a Gram-negative bacterium that has gained particular notoriety as one of the leading causes of nosocomial infections, principally amongst immunocompromised individuals [1]. Similarly, *Ab* is a primary agent for community-acquired pneumonia (CAP), particularly in individuals with a history of alcohol (EtOH) abuse who characteristically present a fulminant clinical course with secondary bloodstream infection and a >50% mortality rate in tropical and sub-tropical climates [2]. Approximately 10% of alcoholics transiently carry this bacterium in the nasopharynx which may act as the source of infection [3]. To exacerbate the problem, *Ab* has an intrinsically extraordinary ability to develop resistance to commonly used antibiotics [4], [5], [6].

Excess EtOH consumption may lead to host's innate and acquired immune deficiency, causing increased susceptibility to infections [7], [8]. For instance, pneumonia mediated by *Klebsiella* or *Streptococcus pneumoniae* augmented mortality in EtOH-treated rodents [9], [10]. Acute and chronic EtOH abuse alters the capacity of monocytes to present antigens to T-cells [11] as well as adversely modifying the levels of pro-inflammatory cytokines produced by phagocytes [12]. Notably, natural killer cell proliferation and activity are considerably reduced after EtOH consumption [13]. Moreover, EtOH exposure elevates serum IgG and IgA antibody levels, a clear indication of B-cell dysfunction [14]. Furthermore, inflammatory mediators are negatively regulated by immune cells following EtOH exposure [15].

Neutrophils circulate in the blood and are among the first cells to arrive at the site of infection; thus, neutrophils play an important role in early modulation of immune response to multi-drug resistant *Ab* infection and tissue injury [16], [17]. We have recently shown the detrimental effects of EtOH on macrophages in *Ab* infection [18]. However, there is lack of information about EtOH effects on specific effector functions of neutrophils in *Ab*-mediated pneumonia. In this study, we hypothesized that physiological EtOH levels impair neutrophil antimicrobial responses enhancing *Ab* pathogenesis. We demonstrated that EtOH damages neutrophil effector functions, an important factor that might enhance the severity of community-acquired *Ab* pneumonia in alcoholic patients resulting in high mortality.

To our knowledge, this is the first study describing the role of EtOH specifically on the setting of *Ab* infection using a mammal experimental infection model. Furthermore, we explore the impact of EtOH on neutrophils which play a critical role in host resistance to respiratory *Ab* infection.

## Materials and Methods

### Bacteria

*Ab* 0057, a clinical isolate acquired from Mark D. Adams (Cleveland, OH), was chosen for this study because it has been sequenced and it is resistant to β-lactam antibiotics including carbapenems but remain susceptible to tigecycline and colistin [19], [20]. The strain was collected from the bloodstream of a soldier in 2004 at Walter Reed Army Medical Center, Washington DC. The isolate was stored at −80°C in Tryptic Soy Broth (TSB; Difco Laboratories, Detroit, MI) with 50% glycerol. Frozen stocks were grown in TSB with rotary shaking at 150 rpm overnight at 37°C. Optical density (OD) measurements were taken at 600 nm (Bio-Tek, Winooski, VT) to monitor growth.

### EtOH administration and infection

To investigate the effects of EtOH in *Ab* pneumonia, 100 µl of 25 mM of EtOH (correspond to 12% in human blood) (Sigma, St. Louis, MO) or Phosphate-buffered saline (PBS; untreated controls) was intraperitoneally (i.p.) injected to female C57BL/6 mice (age, 6–8 weeks; Charles Rivers, Wilmington, MA) daily for 7 days before infection. Sub-lethal infections were induced by and intranasal (i.n.) inoculation of 10^7^ *Ab* cells after anesthetization (ketamine [100 mg/Kg; Ketaset, Fort Dodge, IA]-xylazine [20 mg/Kg; AnaSed, Shenandoah, IA] cocktail). For survival studies, lethal infection was performed by i.n. inoculation with 5×10^7^ *Ab* cells. To monitor the impact of EtOH in mouse survival, EtOH-injected and uninfected animals were used as additional controls.

### Ethics statement

All animal studies were conducted according to the experimental practices and standards approved by the Institutional Animal Care and Use Committee (IACUC) at Long Island University (Protocol #: 11–3). The IACUC at Long Island University approved this study.

### Colony forming units (CFU) determinations

At indicated time points (4, 24, 48, and 72 h) post-infection, mouse tissues (lungs, liver, and kidney) were excised, perfused and externally washed with sterile PBS, before being finally homogenized in PBS. Serial dilutions of homogenates were performed, with 100 µL of each sample plated on TS agar (TSA; Difco) plates and incubated at 37°C for 24 h. Subsequently, bacterial colonies were counted and the results were normalized by tissue weights.

### Cytokine and myeloperoxidase (MPO) determinations

Three mice per group were sacrificed at 4, 24, 48, and 72 h post-infection. The organs of each mouse were excised, perfused and externally washed with PBS, and finally homogenized in PBS with protease inhibitors (Complete Mini; Roche, Ridgefield, CT). Cell debris was removed from homogenates by centrifugation at 6,000 g for 10 min. Samples were stored at −80°C until tested.

- ***Cytokines*** **.** Supernatants were tested for IFN-γ, TNF-α, IL-1β, and IL-6 by ELISA (Becton Dickinson Biosciences, San Diego, CA). The limits of detection were 31.3 pg/mL for IFN-γ and 15.6 pg/mL for TNF-α, IL-1β, and IL-6.
- ***Myeloperoxidase (MPO)*** **.** Supernatants were tested for myeloperoxidase (MPO) by ELISA (Hycult Biotechnology, The Netherlands). The limits of detection were 1 ng/mL for MPO.

### Histological processing

At indicated time-points (4, 24, 48, and 72 h) post*-*infection, organs (lung, liver, and kidney) were excised and fixed in 4% paraformaldehyde for 24 h. Tissues were processed, embedded in paraffin, and 4 µm vertical sections were fixed to glass slides. Tissue sections were stained with Hemotoxylin and Eosin (H&E), Gram or MPO to assess morphology, bacteria, or neutrophil infiltration, respectively. Microscopic examinations of tissues were performed by light microscopy with an Axiovert 40 CFL inverted microscope (Carl Zeiss, Thornwood, NY) and photographed with an AxioCam MrC digital camera using the Zen 2011 digital imaging software (Carl Zeiss).

### Isolation of peripheral blood human neutrophils

Whole human blood was purchased from the Interstate Blood Bank, Inc. (Memphis, TN). Upon arrival, blood cells were pelleted, and erythrocytes removed by hypotonic lysis. Neutrophils were separated from the remaining cells by centrifugation over discontinuous Percoll gradients at 500×*g* for 30 min at 4°C, consisting of 75% (vol/vol) Percoll in PBS. Neutrophils were >95% viable as determined by Wright-Giemsa staining. Recovered neutrophils (∼98% as determined by fluorescence-activated cell sorting (FACS) using Ly-6G as a marker) were cultured (<30 min) in (37°C, 5% CO_2_) in RPMI 1640 (Cellgro, Manassas, VA) supplemented with 10 mM HEPES (pH 7.4) and 10% fetal calf serum (FCS) (Atlanta Biologicals, Lawrenceville, GA) prior to use.

### Neutrophil counts in blood

Seven days after EtOH administration neutrophil counts were performed by differential leukocyte count in all experimental animals using a Hema 3 Stat Pack kit (Fisher Scientific, Hanover Park, IL) and light microscopy.

### Chemotaxis assays

Chemotaxis was measured using a transwell chamber with 6.5 mm diameter polycarbonate filters (3 µm pore size; Corning, Tewksbury, MA). Immediately after isolation, cells were incubated in RPMI 1640 supplemented with FCS in the absence or presence of EtOH (6.25 and 12.5 mM correspond to 3 and 6% of EtOH in human blood, respectively) for 2 h. Then, cells were transferred to RPMI 1640 supplemented with FCS, cultivated on filters and allowed to migrate toward the chemo attractant fMLF (formyl-methionyl-leucyl-phenylalanine, 10^−6^ M; Sigma) or medium alone at 37°C, 5% CO_2_. After 1 h, the filters were removed, and the cells that migrated through the membrane were fixed, stained and counted using light microscopy.

### Phagocytosis assay

Phagocytosis was determined by FACS analysis. Human neutrophils (10^6^ cells) were incubated on 6 well plates with feeding medium supplemented with EtOH (6.25 or 12.5 mM; Sigma), or PBS for 2 h at 37°C and 5% CO_2_. FITC (Molecular Probes, Grand Island, NY)-labeled *Ab* cells were incubated with 25% human serum for 30 min to allow complement proteins to opsonize *Ab*. Bacterial cells were washed and then 10^7^ bacterial cells were added to the 10^6^ neutrophils for 2 h. Similarly, extracellular bacteria were quenched with trypan blue to prevent interference with the assay. Samples were processed (10,000 events per condition) on a LSRII flow cytometer (Becton Dickinson Biosciences, San Diego, CA) and were analyzed using FlowJo software.

### *Ab* killing assay

Since EtOH reduces *Ab* phagocytosis by neutrophils, leukocytes were first allowed to phagocytize *Ab* cells for 0.5 h to determine the initial uptake. Each well containing interacting cells was gently washed with feeding media and incubated with feeding media supplemented with amikacin (200 µg/mL; to kill extracellular bacteria) and either PBS or EtOH (6.25 and 12.5 mM) for 4 h. Viable bacteria were released from neutrophils following 0.5 and 4 h of host-cell interaction by forcibly subjecting culture to a 27-gauge needle passage 5–7 times for efficient lysis [21]. Four microtiter wells per condition were used to ascertain CFU. For each well, serial dilutions were plated in triplicates onto TSA plates, which were incubated at 37°C for 24 h prior to CFU tallying.

### Nitric oxide determinations (NO)

Nitric oxide (NO) produced in supernatants by untreated or EtOH-treated neutrophils was quantified after exposure to *Ab* using a Griess method kit (Promega, Fitchburg, WI).

### Luminol chemiluminescence assay

Reactive oxygen species (ROS) signals were made chemiluminescent (CL) by luminol (1 mM). CL was monitored for 30 min using the automatic luminescence analyzer SpectraMax L (Molecular Devices, Sunnyvale, CA) at 37°C.

### Statistical Analysis

Data were analyzed using Prism (GraphPad, LaJolla, CA). Differences in survival rates were analyzed by the log-rank test (Mantel-Cox). Analysis of cytokine, MPO, chemotaxis, CFU, NO, and chemiluminescence data was done using analysis of variance and adjusted by use of the Bonferroni correction. Analysis of neutrophil count data was performed using student's *t*-test. *P* values of <0.05 were considered significant.

## Results

### EtOH-treated mice display reduce survival in *Ab* infection

**Figure 1:**
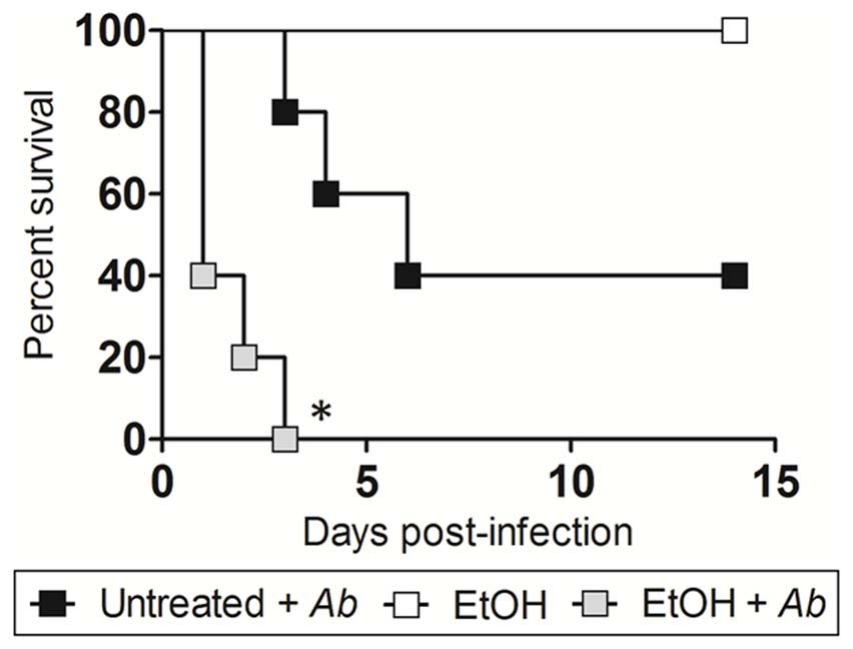
Alcohol (EtOH) administration reduces survival in C57BL/6 mice. Survival differences of EtOH and PBS-injected (Untreated) C57BL/6 mice after infection with 5×10^7^ *Ab* (n = 5 per group). EtOH-treated and uninfected mice were use as additional controls. Asterisks denote *P*-value significance (*P<0.01) calculated by Log-Rank (Mantel-Cox) analysis. This experiment was performed twice and similar results were obtained.

Administration of EtOH accelerated the death of *Ab-*infected mice relative to control mice (*P*<0.01; Fig. 1). On day 3 post-infection, 100% of EtOH-treated mice had died in comparison to 20% of the untreated mice. On average, EtOH-treated mice died of *Ab*-mediated pneumonia 2 days post-infection compared to 9 days for untreated mice (Fig. 1). None of the animals in the EtOH-uninfected group died (Fig. 1). Furthermore, similar to humans who abuse EtOH, treated animals displayed marked stereotypic behaviors such as loss of motor coordination and distress. These behaviors were most apparent 5–10 min after EtOH injection and continued for several hours.

### EtOH exacerbates *Ab*-mediated pneumonia

**Figure 2:**
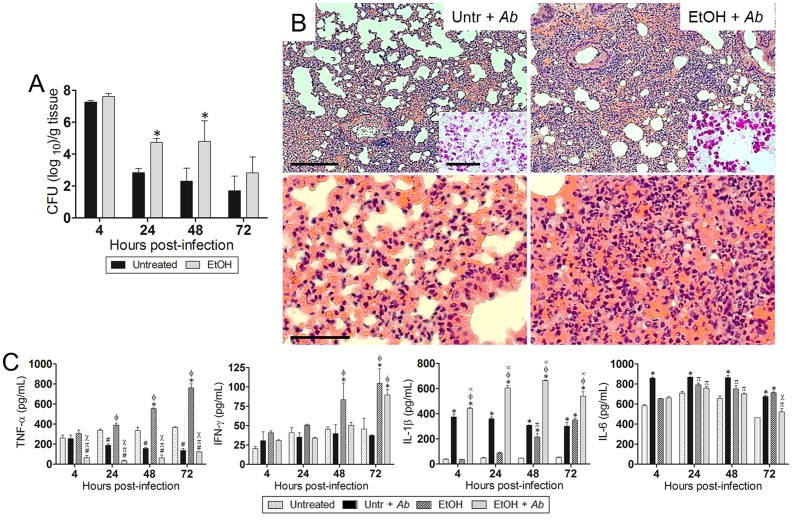
EtOH exacerbates *Ab*-mediated pneumonia *in vivo*. (**A**) Bacterial burden (CFU; colony forming unit) in lungs excised from untreated and EtOH-treated mice, infected with 10^7^ *Ab* cells (*n* = 3 per group) 4, 24, 48, and 72 h post-infection. Solid and error bars denote the means and standard deviations, respectively. Asterisks denote *P*-value significance (*P<0.05) calculated by analysis of variance (ANOVA) and adjusted by use of the Bonferroni correction. This experiment was performed twice and similar results were obtained. (**B**) Histological analysis of lungs removed from untreated-*Ab*-infected (Untr + *Ab*) and EtOH-treated-*Ab*-infected (EtOH + *Ab*) C57BL/6 mice 48 h post-infection. Representative low (10X; scale bar: 20 µm) and high (40X; scale bar: 10 µm) magnification H&E-stained sections of the lungs are shown with Gram staining *insets* (upper panel; Scale bars: 10 µm) showing the gram-negative *Ab*. (**C**) Cytokines levels (TNF-α, IFN-γ, IL-1β, and IL-6; pg/mL) in the lungs of C57BL/6 mice. Solid and error bars denote the means and standard deviations, respectively. Symbols denote *P*-value significance (*P*<0.05) calculated by ANOVA and adjusted by use of the Bonferroni correction. *, φ, ∞ indicate higher levels than untreated, untr + *Ab*, EtOH groups, respectively. #, π, χ indicate lower levels than untreated, untr + *Ab*, EtOH groups, respectively. These experiments were performed twice and similar results were obtained.

In sub-lethally infected mice, pulmonary bacterial burden of EtOH-treated animals was significantly higher than untreated mice (*P*<0.05; Fig. 2A) 24 and 48 h after *Ab* infection. Histological examination revealed that untreated-uninfected and EtOH-treated-uninfected mice had minimal or no inflammation (data not shown). After infection, untreated and EtOH-treated mice had significant peribronchial inflammation and inflammatory cells present within the alveoli (Fig. 2B). Gram staining showed gram negative bacterial cells within the alveolar spaces of the lungs of infected animal either untreated or EtOH-treated (Fig. 2B; *insets*; upper panel). In summary, these studies demonstrated that EtOH administration enhances disease progression by showing greater pulmonary *Ab* burden compared to the untreated group as shown in the CFU data. We measured pro-inflammatory cytokine response (TNF-α, IFN-γ, IL-1β, and IL-6) in the lungs of untreated or EtOH-treated mice and uninfected or infected with *Ab* at 4, 24, 48, and 72 h post-infection (Fig. 2C). The pulmonary tissue of infected mice treated with EtOH and infected with *Ab* contained significantly higher quantities of IL-1β (*P*<0.05) and reduced levels of TNF-α (*P*<0.05) in contrast to other group conditions. The untreated-*Ab*-infected group displayed significantly lower levels of TNF-α (*P*<0.05) than untreated controls 24 h post-infection. EtOH-treated animals showed the highest levels of TNF-α (*P*<0.05) at 24–72 h. Similarly, EtOH-treated animals exhibited the highest levels of IFN-γ (*P*<0.05) at 48 and 72 h whereas this cytokine was only significantly elevated in EtOH-treated-*Ab*-infected mice 72 h post-infection. Lastly, untreated-*Ab*-infected group evinced the highest levels of IL-6 production (24–48 h; *P*<0.05), although EtOH-treated group showed a similar increase in this cytokine at 72 h.

### EtOH accelerates *Ab* dissemination from the lungs to other organs

**Figure 3:**
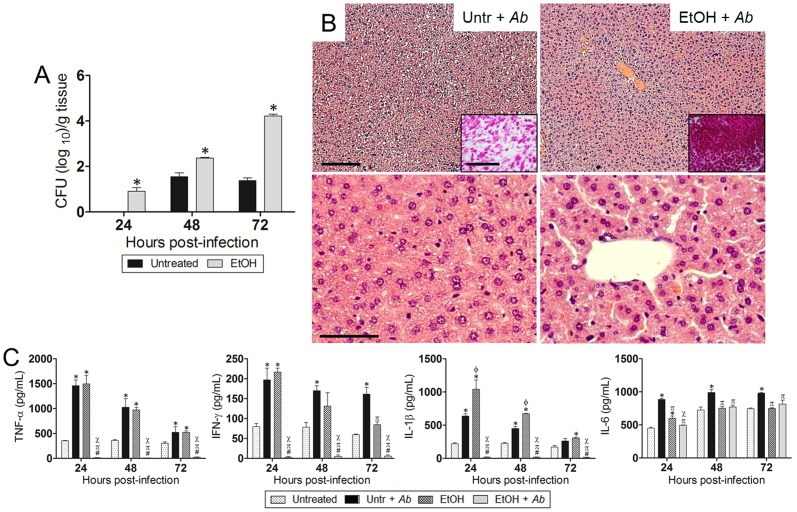
EtOH accelerates *Ab* dissemination from the lungs to the liver after 24 h of infection. (**A**) Bacterial burden (CFU) in liver (24, 48, and 72 h post-infection) excised from untreated and EtOH-treated mice, infected with 10^7^ *Ab* 0057 strain (*n* = 3 per group). Solid and error bars denote the means and standard deviations, respectively. Asterisks denote *P*-value significance (*P<0.01) calculated by ANOVA and adjusted by use of the Bonferroni correction. This experiment was performed twice and similar results were obtained. (**B**) Histological analysis of liver removed from untreated- and EtOH-treated-*Ab*-infected C57BL/6 mice. Representative low (10X; scale bar: 20 µm) and high (40X; scale bar: 10 µm) magnification H&E-stained sections of the liver are shown with Gram staining *insets* (upper panel; Scale bars: 10 µm) showing the gram-negative *Ab* (72 h post-infection). (**C**) Cytokines levels (TNF-α, IFN-γ, IL-1β, and IL-6; pg/mL) in the liver of C57BL/6 mice. Solid and error bars denote the means and standard deviations, respectively. Symbols denote *P*-value significance (*P*<0.05) calculated by ANOVA and adjusted by use of the Bonferroni correction. *, φ, ∞ indicate higher levels than untreated, untr + *Ab*, EtOH groups, respectively. #, π, χ indicate lower levels than untreated, untr + *Ab*, EtOH groups, respectively. These experiments were performed twice and similar results were obtained.

**Figure 4:**
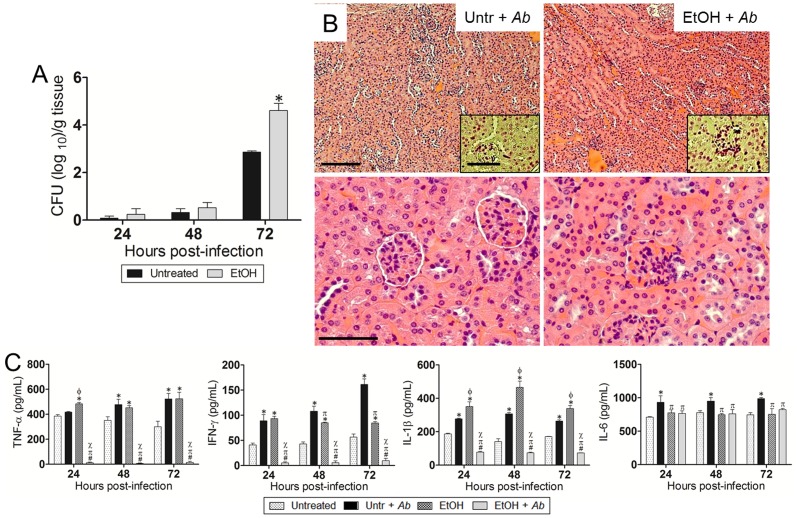
EtOH enhances *Ab* dissemination from the lungs to the kidney 72 h post-infection. (**A**) Bacterial burden (CFU) in kidneys (24, 48, and 72 h post-infection) excised from untreated and EtOH-treated mice, infected with 10^7^ *Ab* 0057 strain (*n* = 3 per group). Solid and error bars denote the means and standard deviations, respectively. Asterisks denote *P*-value significance (*P<0.01) calculated by ANOVA and adjusted by use of the Bonferroni correction. This experiment was performed twice and similar results were obtained. (**B**) Histological analysis of kidneys removed from untreated- and EtOH-treated-*Ab*-infected C57BL/6 mice. Representative low (10X; scale bar: 20 µm) and high (40X; scale bar: 10 µm) magnification H&E-stained sections of the kidneys are shown with Gram staining *insets* (upper panel; Scale bars: 10 µm) showing the gram-negative *Ab* (72 h post-infection). (**C**) Cytokines levels (TNF-α, IFN-γ, IL-1β, and IL-6; pg/mL) in the kidneys of C57BL/6 mice. Solid and error bars denote the means and standard deviations, respectively. Symbols denote *P*-value significance (*P*<0.05) calculated by ANOVA and adjusted by use of the Bonferroni correction. *, φ, ∞ indicate higher levels than untreated, untr + *Ab*, EtOH groups, respectively. #, π, χ indicate lower levels than untreated, untr + *Ab*, EtOH groups, respectively. These experiments were performed twice and similar results were obtained.

We investigated whether EtOH enhances *Ab* dissemination from the lungs to other organs. *Ab* significantly disseminated from the lungs of EtOH-treated mice to the liver in 24 h (*P*<0.05; Fig. 3A) and to the kidneys in 72 h (*P*<0.05; Fig. 4A) after intranasal infection. Histological analysis revealed hepatocellular atrophy (Fig. 3B) and considerable presence of bacteria in the livers of animals treated with EtOH (Fig. 3B; *insets*; upper panel). We evaluated the levels of TNF-α, IFN-γ, IL-1β, and IL-6 in the hepatic tissue of untreated or EtOH-treated mice and uninfected or infected with *Ab* at 24, 48, and 72 h post-infection (Fig. 3C). The liver of infected animals treated with EtOH and infected with *Ab* showed significantly lower levels of TNF-α (*P*<0.05), IFN-γ (*P*<0.05), and IL-1β (*P*<0.05) than those of the other experimental conditions. Untreated-*Ab*-infected and EtOH-treated animals showed significant increase in TNF-α (*P*<0.05), IFN-γ (*P*<0.05), and IL-1β (*P*<0.05) levels compared to untreated controls. Also, these three cytokines were highly elevated 24 h after *Ab* infection in untreated-*Ab*-infected and EtOH-treated groups but gradually reduced at 48 and 72 h. Untreated-*Ab*-infected mice showed the highest levels of IL-6 production (*P*<0.05). EtOH and *Ab*-infected animals demonstrated lower IL-6 levels than EtOH-treated animals (*P*<0.05) 24 h post-infection.

Kidneys excised from EtOH-treated animals presented nephromegaly with abnormally thickened basement membrane of the glomeruli and cell proliferation (Fig. 4B). Additionally, high bacterial burden was observed in EtOH-treated mice, mostly accumulated in the glomeruli of the kidneys (Fig. 4B; *insets*; upper panel). Untreated mice displayed uniform bacterial cell distribution throughout the cortex and medulla of this excretory organ (Fig. 4B; *inset*; upper panel). We examined the levels of TNF-α, IFN-γ, IL-1β, and IL-6 in the kidneys of untreated or EtOH-treated mice and uninfected or infected with *Ab* at 72 h post-infection (Fig. 4C). The renal tissue of infected mice treated with EtOH and infected with *Ab* contained significantly reduced levels of TNF-α (*P*<0.05), IFN-γ (*P*<0.05), and IL-1β (*P*<0.05) compared to the other experimental conditions. Untreated-*Ab*-infected and EtOH-treated animals exhibited significant increase in TNF-α (*P*<0.05) and IL-1β (*P*<0.05) levels compared to untreated controls. In addition, significant increases in IL-6 production (*P*<0.05) were observed in untreated-*Ab*-infected mice compared to the other conditions.

### EtOH enhances neutrophil infiltration

**Figure 5:**
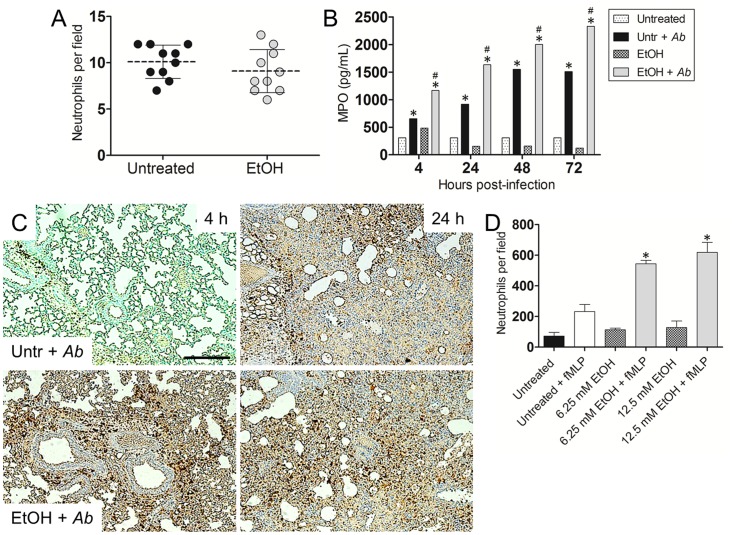
EtOH administration and *Ab* infection increases pulmonary neutrophil infiltration. (**A**) Number of neutrophils per field in blood of untreated or EtOH-treated mice. Each black circle or red square represents the numbers of phagocytes per individual field. Dashed lines and error bars denote average of ten counts and standard deviations, respectively. *P*-value significance (*P*<0.05) was calculated using student's *t*-test analysis. (**B**) Pulmonary myeloperoxidase (MPO) levels. After EtOH administration and *Ab* infection, homogenates of extracted lungs from C57BL/6 mice were prepared and the supernatants were analyzed for MPO levels. * and **#** indicates increased MPO levels compared to untreated and untreated-Ab-infected (Untr + *Ab*) groups, respectively. (**C**) Histological analysis of lungs removed from untreated-*Ab* infected and EtOH-treated-*Ab*-infected C57BL/6 mice. Representative MPO-stained sections show neutrophil infiltration (brown staining). Scale bars: 10 µm. (**D**) Human neutrophil migration after exposure of uninfected cells to the chemoattractant fMLF. Peripheral neutrophils were isolated from human blood and treated with PBS (untreated) or EtOH (6.25 or 12.5 mM). Neutrophils unexposed to fMLF were used as controls. For (**B**) and (**D**), solid and error bars denote the means and standard deviations, respectively. Asterisks denote *P*-value significance (*, #*P*<0.001) calculated by ANOVA and adjusted by use of the Bonferroni correction. These experiments were performed twice and similar results were obtained.

We examined whether EtOH administration affected the number of circulating neutrophils in the blood of C57BL/6 mice using differential leukocyte staining. Cell count analysis showed that EtOH-treated animals had no difference in blood circulating phagocytes when compared to controls (Fig. 5A).

MPO is highly produced by neutrophils, therefore, detection of this enzyme is commonly used as a surrogate to quantify neutrophil recruitment to different tissues. MPO yields hypochlorous acid from hydrogen peroxide and chloride anion during the neutrophil's respiratory burst. Therefore, we assessed the effect of EtOH on neutrophil recruitment in the lungs of C57BL/6 mice by neutrophils. Mice treated with EtOH (*P*<0.001) or untreated (*P*<0.001) resulted in significantly higher levels of MPO after *Ab* infection than did untreated neutrophils (Fig. 5B). Similarly, we identified neutrophil infiltration using immunohistochemistry (IHC) by measuring the expression of MPO in pulmonary tissue. Tissue sections from EtOH murine lungs infected with *Ab* exhibited early (4 h) and massive neutrophil infiltrations when compared to lungs excised from untreated-infected animals (Fig. 5C). Additionally, uninfected lungs demonstrated minimal neutrophil infiltration (data not shown). To confirm the IHC findings, EtOH was tested for its ability to promote human neutrophil chemotaxis *in vitro*. EtOH exposure (6.25 mM; *P*<0.001 and 12.5 mM; *P*<0.001) significantly stimulated higher leukocyte migration than untreated or fMLF-treated controls (Fig. 5D).

### EtOH reduces neutrophils phagocytosis and killing of *Ab*

**Figure 6:**
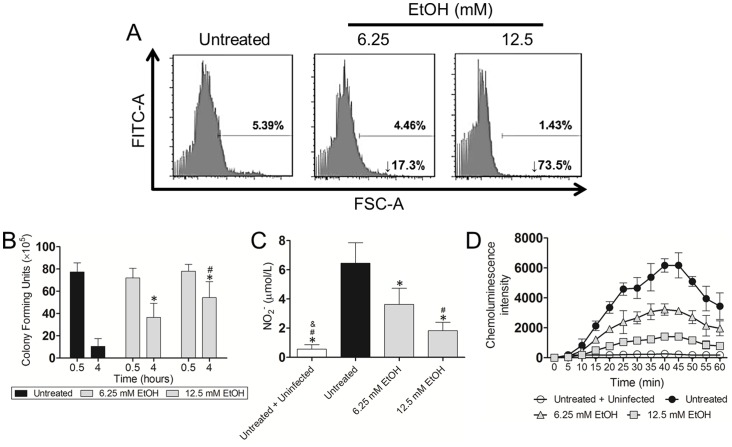
EtOH reduces human neutrophil phagocytosis, nitric oxide production, and killing of *Ab*. Neutrophils were untreated or exposed to EtOH for 2*Ab*. (**A**) Phagocytosis of FITC-labeled *Ab* by human neutrophils was determined using Fluorescent Activated Cell Sorting (FACS) analysis. Representative histograms are shown. (**B**) Killing of *Ab* by neutrophils was determined using colony-forming units (CFU) analysis. Solid and error bars denote means and standard deviations, respectively. Symbols denote *P*-value significance (*P*<0.05) calculated by ANOVA and adjusted by use of the Bonferroni correction. * and # indicate higher CFU numbers than untreated and 6.25 mM EtOH groups, respectively. (**C**) Nitric oxide (NO) production was quantified using the Griess method after untreated or EtOH-treated neutrophils were co-incubated with *Ab*. Untreated and uninfected neutrophils were also used as controls. Solid and error bars denote means and standard deviations, respectively. Symbols denote *P*-value significance (*P*<0.05) calculated by ANOVA and adjusted by use of the Bonferroni correction. *, #, & indicate lower levels than untreated, 6.25 and 12.5 mM EtOH groups, respectively. (**D**) Oxidative burst was quantified for 60 min using luminol chemiluminescence after untreated or EtOH-treated neutrophils were co-incubated with *Ab*. Untreated and uninfected neutrophils were also used as controls. Symbols and error bars denote means and standard deviations, respectively. *P*-value significance (*P*<0.05; discussed in result section) was calculated by ANOVA and adjusted by use of the Bonferroni correction at each time point. For (**A**)–(**D**), experiments were performed twice and similar results were obtained.

We analyzed the effects of physiological EtOH on *Ab* phagocytosis and killing by neutrophils using FACS analysis. EtOH reduced phagocytosis of *Ab* by neutrophils, compared with the control (Fig. 6A). Our results showed a 17.3 and 73.5% phagocytosis inhibition in cells treated with 6.25 and 12.5 mM EtOH, respectively, when compared to control cells. We examined whether EtOH interferes with neutrophil-mediated killing of *Ab* cells. EtOH significantly reduced bacterial killing by human neutrophils (*P*<0.05) (Fig. 6B). Consequently, we investigated the impact of EtOH on extracellular NO production by these leukocytes after co-incubation with *Ab*. Our results indicate that NO levels were significantly reduced in the supernatants of EtOH-treated cells (6.25 mM; *P*<0.05 and 12.5 mM; *P*<0.001) when compared to controls (Fig. 6C). Finally, neutrophils mostly kill bacteria via NADPH oxidase-derived ROS. Hence, we evaluated the impact of EtOH on neutrophils' oxidative burst by measuring luminol chemiluminescence intensity. Increased concentration of EtOH significantly decreased ROS production compared to untreated-*Ab* neutrophils (*P*<0.05; 15 to 60 min). Minimal production of ROS was observed in untreated and unstimulated control cells.

## Discussion

EtOH abuse has been previously shown to predispose the host to CAP, particularly to multi-drug resistant *Ab* resulting in significant illness and mortality [2]. In this study, we demonstrated that EtOH administration had a profound effect on survival in mice i.n. challenged with *Ab*. The increased mortality in EtOH-treated mice was attributable to the inability of immune cells to clear infection. We observed a significant increase in bacterial burden in the lungs of EtOH-treated animals, compared to controls. After infection and in contrast to untreated animals, EtOH-treated mice displayed high inflammation and number of inflammatory cells present within the alveoli suggesting that increased *Ab* burden and reduced animal survival were not attributable to a diminished recruitment of immune cells to the lungs, but to decreased cellular microbicidal capacity. In this regard, high levels of IL-1β present in the pulmonary tissue of EtOH-treated mice suggest that this cytokine compensates the late production of TNF-α and IFN-γ. In contrast, untreated-*Ab*-infected mice displayed early high levels of TNF-α followed by a time-dependent reduction of this cytokine and elevated levels of IL-1β and IL-6 which may explain reduced bacterial burden in these animals. Another contributing factor to the inability of treated mice to reduce bacterial numbers is that EtOH changes pulmonary surfactant production which may result in a lessening antibacterial activity [22].

MPO IHC demonstrated that EtOH-treated animal experienced earlier and higher recruitment of neutrophils to the lungs post-infection than the untreated-*Ab*-infected group. To confirm the IHC results, quantitative analysis exhibited a considerable time-dependent increased and sustained MPO production in pulmonary tissue of EtOH-treated animals. Perhaps, EtOH stimulates early and uncontrolled massive recruitment of neutrophils to the infection site increasing the probability of tissue damage by activated leukocytes. This observation is also supported by dysregulated levels of pro-inflammatory cytokines found in lung homogenates of EtOH-treated mice. For instance, neutrophil apoptosis might be impaired extending the presence of these phagocytic cells in lung tissue which might be detrimental for the host's tissue architecture [23], [24]. Consistent with previous studies, our findings indicated that EtOH administration did not reduce neutrophil infiltration to the infection site [25], which might suggest that this substance of abuse impairs the ability of phagocytic cells to engulf and kill *Ab* within the lungs [26], [27], resulting in early dissemination of infection to the bloodstream and other organs. For example, human neutrophils treated with physiological levels of EtOH showed increased chemotaxis *in vitro* and decreased *Ab* phagocytosis and killing. Similarly, we have recently shown that EtOH-mediated phagocytosis dysfunction may be associated with reduced expression of GTPase-RhoA, a key regulator of the actin polymerization signaling cascade using a murine J774.16 macrophage-like cell line [18].

After phagocytosis, bacteria are rapidly exposed to the microbicidal armamentarium conferred by neutrophils, which consist of toxic reactive species, such as NO, and lysosomal hydrolases. Our data show that NO generation is significantly decreased in neutrophils that are exposed to EtOH. This phenomenon might be explained by reduction in TNF-α levels. Impaired production of NO and ROS might create an ideal environment for microbial survival, facilitating intracellular replication and dysregulation of the phagolysosomal milieu. For instance, EtOH reduced NO production by alveolar macrophages after challenge with *Mycobacterium tuberculosis* [28]. Recent work in our laboratory demonstrated that inducible NO synthase expression is reduced after macrophage-like cells were exposed to EtOH [18].

Liver damage is common in EtOH users, often leading to hepatitis, cirrhosis, and fatty liver. The accumulation of EtOH in the liver may be responsible for the hepatocellular atrophy we observed in EtOH-treated animals. One of the potential mechanisms by which EtOH can cause exacerbation of *Ab* infection is, in part, linked to the deleterious interaction of EtOH and bacterial lipopolysaccharide (LPS) on the immune response [29]. Thus, EtOH induced immunosuppression may be promoting this gram negative organism's dissemination and replication within hepatic tissue. Surprisingly, we observed reduced levels of TNF-α, which have been previously implicated with cell death and liver damage in alcoholics when overproduced by Kupffer cells [30], [31], [32]. Our findings provide fundamental insights into how EtOH may play an important role in *Ab* infection-related morbidity and mortality in a vertebrate model of infection.

Chronic alcohol consumption can cause kidney dysfunction, mainly in conjunction with established liver disease. Excised kidneys from EtOH-treated mice exhibited higher *Ab* burden and swelling than controls at 72 h post-infection. Bacterial distribution in the renal tissue was also different with *Ab* accumulation in the glomeruli of EtOH-treated mice as opposed to uniform dispersed bacteria throughout the organ observed in the control animals. This result suggests that EtOH predispose alcoholics to urinary tract infections and consequent kidney failure which may result in death [33], [34]. This clinical scenario is complicated by the multi-drug resistance capacity of *Ab* and reduced pro-inflammatory cytokine production, increasing the host's susceptibility to an adverse prognosis. Similarly, kidney enlargement in alcoholics is of clinical significance.

Although Cheung et al., have previously shown that the differential count for the neutrophils in young and mature rats treated with alcohol is lower than in untreated animals [35]. In humans, various abnormalities in circulating neutrophils also have been described with alcohol consumption, ranging from an increase in the number of these cells in the peripheral blood to neutropenia in those with the most severe form of infection or severe underlying hepatic disease [36]. We found that EtOH-treated animals had no differences in blood circulating neutrophils when compared to untreated controls further supporting the notion that *Ab* infection could not be controlled in EtOH-treated animals due to a reduction of the antimicrobial effector functions of these leukocytes.

Previous studies have shown that EtOH exposure may increase *Ab* virulence. For instance, EtOH promotes secretion of the outer membrane protein OmpA, which is important in *Ab* biofilm formation and induces epithelial cell apoptosis [37], [38], [39]. Similarly, co-culture of *Ab* with the baker's yeast *Saccharomyces cerevisiae* promoted bacterial growth, primarily due to fungal-mediated EtOH production [40]. Perhaps, EtOH provides *Ab* with the capacity to tolerate salt stress, making *Ab* an extremely successful opportunistic pathogen for persons abusing alcohol who copiously sweat due to increased body heat. *Ab* genomic and proteomic analyses revealed that EtOH regulates genes responsible for stress responses, drug resistance, iron transport, and biofilm formation [41], [42]. Furthermore, EtOH enhances *Ab* pathogenesis in invertebrate models of infection [42], [43], [44].

The majority of CAP-*Ab* infections occur in individuals with underlying comorbidities, who reside in tropical and subtropical climates [45]. For instance, Australians aborigines in the Northern Territory are overrepresented relative to the general population in rates of CAP caused by *Ab* [46]. This disparity has been attributed to the interaction of both climate and a high prevalence of comorbidities in the indigenous Australian population including alcoholism, diabetes mellitus, chronic obstructive pulmonary disease and cigarette smoking [46], [47]. Although it is not clear whether immune cell dysfunction directly contributes to CAP *Ab* infection in humans, our study revealed that EtOH administration is positively associated with the progression of this infection in an animal model. Moreover, alcohol consumption has been previously correlated with impaired immune responses including alveolar macrophages dysfunction in phagocytosis, killing of bacteria, and cytokine secretion [46].

It is important to mention that CAP-*Ab* is almost never detected in countries outside the tropics despite the fact that alcohol abuse is common in many countries around the world. It is possible that tropical regions are favorable and optimal for the growth of *Ab* in the environment. For instance, *Ab* is a very resilient microbe resistant to high temperature (≥47°C) and desiccation [48]. Nevertheless, correlating climate and infection is out of the scope of this study, a very interesting question to pursue in future studies.

In conclusion, this is the first report that experimentally demonstrates that EtOH intensifies *Ab*-associated pneumonia and disease progression *in vivo* by deregulating neutrophil antimicrobial functions. The synthesis of these findings, including recent advances in *Ab* virulence studies, should raise awareness on the negative impact of EtOH abuse in alcoholics, specifically in prevalent regions. The ability of *Ab* to cause disease in alcoholics underscores how the study of its virulence mechanisms and host interactions is necessary.
